# Misattributing the Source of Self-Generated Representations Related to Dissociative and Psychotic Symptoms

**DOI:** 10.3389/fpsyg.2016.00541

**Published:** 2016-04-21

**Authors:** Chui-De Chiu, Mei-Chih Meg Tseng, Yi-Ling Chien, Shih-Cheng Liao, Chih-Min Liu, Yei-Yu Yeh, Hai-Gwo Hwu

**Affiliations:** ^1^Department of Psychology, The Chinese University of Hong KongHong Kong SAR, The People's Republic of China; ^2^Department of Psychology, National Taiwan UniversityTaipei, Taiwan; ^3^Department of Psychiatry, National Taiwan UniversityTaipei, Taiwan; ^4^Department of Psychiatry, Far Eastern Memorial HospitalNew Taipei City, Taiwan; ^5^Department of Psychiatry, National Taiwan University HospitalTaipei, Taiwan

**Keywords:** dissociation, generation effect, psychosis, self, source monitoring, trauma

## Abstract

**Objective:** An intertwined relationship has been found between dissociative and psychotic symptoms, as the two symptom clusters frequently co-occur, suggesting some shared risk factors. Using a source monitoring paradigm, previous studies have shown that patients with schizophrenia made more errors in source monitoring, suggesting that a weakened sense of individuality may be associated with psychotic symptoms. However, no studies have verified a relationship between sense of individuality and dissociation, and it is unclear whether an altered sense of individuality is a shared sociocognitive deficit underlying both dissociation and psychosis.

**Method:** Data from 80 acute psychiatric patients with unspecified mental disorders were analyzed to test the hypothesis that an altered sense of individuality underlies dissociation and psychosis. Behavioral tasks, including tests of intelligence and source monitoring, as well as interview schedules and self-report measures of dissociative and psychotic symptoms, general psychopathology, and trauma history, were administered.

**Results:** Significant correlations of medium effect sizes indicated an association between errors attributing the source of self-generated items and positive psychotic symptoms and the absorption and amnesia measures of dissociation. The associations with dissociative measures remained significant after the effects of intelligence, general psychopathology, and trauma history were excluded. Moreover, the relationships between source misattribution and dissociative measures remained marginally significant and significant after controlling for positive and negative psychotic symptoms, respectively.

**Limitations:** Self-reported measures were collected from a small sample, and most of the participants were receiving medications when tested, which may have influenced their cognitive performance.

**Conclusions:** A tendency to misidentify the source of self-generated items characterized both dissociation and psychosis. An altered sense of individuality embedded in self-referential representations appears to be a common sociocognitive deficit of dissociation and psychosis.

## Introduction

An investigation into the relationship between dissociation and psychosis has been revived in recent years (van der Hart and Witztum, 2008; Moskowitz and Heim, 2011; Longden et al., 2012). Dissociation and psychosis are considered two distinct clusters of psychopathology in the Diagnostic and Statistical Manual of Mental Disorders, Fifth Edition (DSM-5). Psychosis is characterized by a lack of reality monitoring with hallucination and delusion, whereas dissociation is characterized by a disruption and/or discontinuity in the normal integration of consciousness, memory, identity, emotion, perception, body representation, motor control, and behavior. Psychotic symptoms and psychotic disorders are not considered associative features or comorbidities of dissociative disorders. However, dissociation has been found to be associated with psychosis (Kluft, 1987; Ross et al., 1990b). Approximately 94% of patients with a dissociative disorder have reported experiencing a Schneiderian first-rank symptom (Spiegel et al., 2013). Positive symptoms including hallucination and delusion have been shown to be correlated with dissociative symptoms (Kilcommons and Morrison, 2005; Vogel et al., 2009). Approximately 15–60% of patients with schizophrenic spectrum disorders have been diagnosed as having a dissociative disorder (Ross and Keyes, 2004; Yu et al., 2010).

This interrelationship suggests that the two clusters of psychopathology may have common risk factors. One environmental factor that has attracted attention is traumatic stress in childhood. Both dissociative and psychotic symptoms have been positively correlated with childhood physical and sexual maltreatment (Dalenberg et al., 2012; Matheson et al., 2013). Less attention has been paid to intrinsic factors, although a distorted perception of individuality appears phenomenally to characterize both symptom clusters (Oyebode, 2008). Individuality is the experience of being indivisible and having a distinct existence from other people and things in the external world. A foundation of this experience involves the ability to discriminate between mental events that have deliberately been constructed by the self and those taking place in response to the environment (Sass and Parnas, 2003). With this ability, the representations of self-initiated events can be differentiated from those driven primarily by the external world, which entails a subjective awareness of the ownership of subsequent emotions, thoughts, memories, and intentions. Clinical observations have shown a diminished sense of individuality in the emotions, thoughts, and actions related to psychotic symptoms (e.g., passivity), and the internal experience originating from the self may be misinterpreted as input imposed from an external source or by an external agent (Keefe, 1998; Nelson et al., 2009). Similarly, a lack of ownership of autobiographical experiences characterizes dissociative amnesia (Markowitsch and Staniloiu, 2011). Clinical observations suggest that a diminished sense of individuality in self-referential representations may be a common intrinsic characteristic of both psychosis and dissociation.

A distorted sense of individuality in self-referential representations has been posited as a vital mechanism of psychotic symptoms (Nelson et al., 2014). This hypothesis has been verified rigorously in patients with schizophrenia spectrum disorders. Sense of individuality in mental events is measured by a verbal self-other source monitoring task (Mitchell and Johnson, 2009). In this task, participants study several sentences with a target item generated either by themselves (e.g., A cat chases a m     ) or acquired from an experimenter (e.g., A rabbit eats a carrot). During the test, participants determine whether a sentence has been seen before (item recognition) and judge whether the target item in the sentence was offered by the experimenter or generated by themselves (source attribution). The results have shown higher rates of source attribution errors in patients with schizophrenia spectrum disorders compared with healthy controls. Some studies have emphasized the misattribution of self-generated items as being offered by others (Vinogradov et al., 1997, 2008; Keefe et al., 2002; Fisher et al., 2008); other studies have viewed misattribution of other-provided items as constructed by the self (Moritz et al., 2003; Wang et al., 2011). As schizophrenia spectrum disorders show two distinct symptom profiles (Crow, 1980; Liddle et al., 1994), the two types of source attribution errors may be associated with the different profiles. Supporting this proposition, studies have shown that schizophrenic patients with hallucination committed more source attribution errors on self-generated items than those without hallucination (Brunelin et al., 2006) and that patients with blunt affect made more source attribution errors on other-provided items (Brébion et al., 2002). Hallucination and delusion characterize positive symptoms, whereas blunted affect, social and emotional withdrawal, and difficulty in abstract thinking characterize negative symptoms.

In contrast with the efforts to verify the association between psychosis and the altered sense of individuality in self-referential representations, little effort has been made to investigate whether dissociative individuals are also biased in source attribution. One study has shown that people with recovered trauma memory, a phenomenon related to dissociation (Chiu et al., 2012b), were inefficient in source attribution between items they had seen on a computer screen and items they had imagined seeing (McNally et al., 2005). Dissociative individuals may be less able to distinguish internal from external representations. It remains to be seen, however, whether deficient source attribution of materials generated by different agents (i.e., another person vs. the self) is associated with dissociation. Additionally, no studies have examined whether potentially traumatizing events can account for the source attribution bias, despite the fact that it has been postulated that early trauma can cause an altered sense of individuality in dissociation and psychosis (van der Hart and Witztum, 2008; Markowitsch and Staniloiu, 2011).

This study investigated the relationship between self-other source monitoring, dissociation, and psychosis. Rather than focusing on a specific disorder, a clinical sample of unspecified psychiatric inpatients was recruited because dissociation appeared to be common co-morbid psychopathology which was under-reported (Saxe et al., 1993; Latz et al., 1995; Lussier et al., 1997; Şar et al., 2000; Foote et al., 2006; Ginzburg et al., 2010). We examined whether the tendency to misattribute the source of self-generated items as obtained from others was related to both dissociative and psychotic symptoms. Assuming that a diminished sense of individuality is an intrinsic sociocognitive factor that underlies both dissociation and psychosis, we hypothesized that source attribution error would be related to both dissociative and psychotic symptoms. Specifically, we hypothesized that source misattribution of self-generated representations would underlie positive psychotic symptoms and dissociative symptoms, as positive symptoms have been correlated with dissociative symptoms (Kilcommons and Morrison, 2005; Vogel et al., 2009). Based on a previous study that showed that misattribution of other-provided items was related to blunt affect in schizophrenic patients (Brébion et al., 2002), we postulated that negative psychotic symptoms would be associated with source misattribution of other-provided representations.

A modified source-monitoring paradigm with self-generated and other-provided items was administered to a sample of unspecified acute psychiatric inpatients. This sample was tested for two reasons. First, an altered sense of individuality reflected in pathological amnesia and psychotic symptoms may be uncommon in nonclinical participants. Second, it was of interest to investigate whether the associations between two distinct symptom profiles of schizophrenia and two types of source attribution errors (Brébion et al., 2002; Brunelin et al., 2006) could be generalized beyond the schizophrenia spectrum disorders. That is, the issue of interest was the interconnected relationship associating source misattribution with dissociative symptoms and psychotic symptoms across a spectrum of various disorders. To characterize the associations, positive and negative psychotic symptoms were measured in addition to two instruments of dissociation. It has been suggested that dissociation may not be unidimensional (Holmes et al., 2005; Nijenhuis and van der Hart, 2011). Some forms of dissociation including absorption and mild gaps in awareness are known to be common in the general population and may not necessarily lead to disability and distress (Ross et al., 1990a; Carlson, 1994). Others such as depersonalization and amnesia are more pathological and prevalent in the clinical population (i.e., Waller et al., 1996). Intellectual function was assessed and controlled for in the analysis, as source misattribution errors in psychiatric patients may have resulted from general cognitive deficiency (Vinogradov et al., 1997). Traumatic experiences in childhood and adulthood as well as global psychopathology were also assessed.

On the basis of empirical evidence obtained from studies on schizophrenia spectrum disorders (Vinogradov et al., 1997, 2008; Brébion et al., 2002; Keefe et al., 2002; Moritz et al., 2003; Brunelin et al., 2006; Fisher et al., 2008; Wang et al., 2011), misattribution of self-generated items was expected to be associated with positive psychotic symptoms and misattribution of other-provided items to be related to negative symptoms. A relationship between source misattribution of self-generated items and amnesia measured by dissociation instruments was expected, as dissociative amnesia is characterized by lacking the ownership of autobiographical experiences (Markowitsch and Staniloiu, 2011). In addition to amnesia, we hypothesized that absorption in dissociation would also be related to attribution error because absorption can blur the boundary between the internal and external worlds. The question of critical importance was whether the relationship between source misattribution and dissociative symptoms would be eliminated after controlling for the influence of psychotic symptoms. This result would shed light on the common and unique sociocognitive mechanisms that underlie dissociative and psychotic symptoms.

Two competing hypotheses existed for the role of traumatic experience in linking source misattribution with dissociative and psychotic symptoms. As a risk factor for both dissociation and psychosis, traumatic stress that occurs during stress-sensitive periods of development can lead to a psychophysiological vulnerability (Heim et al., 2010). This psychophysiological vulnerability has been shown to occur in neural regions pertinent to the processing of self-referential information (van Harmelen et al., 2010), and the development of sense of individuality may therefore be distorted. Accordingly, traumatic stress may be correlated with source misattribution and could mediate its relationship with psychotic and dissociative symptoms. In contrast, source misattribution may be a sociocognitive endophenotype of dissociation proneness. It has been shown that traumatic experiences do not account for the atypical cognitive functions in nonclinical dissociators (DePrince et al., 2008; Chiu et al., 2010, 2012a). Traumatic experiences may therefore not account for the association of source misattribution with dissociative symptoms.

## Methods

### Participants

The study was part of a clinical project of dissociation in acute psychiatric inpatients in Taiwan. The project was approved by the Institutional Review Board at the National Taiwan University Hospital. Participants were recruited from two acute wards. One ward was for deficient reality testing or violent behaviors, mainly with psychotic disorders and bipolar affective disorder (Ward-P); the other was for mood disturbances, anxiety, somatic complaints, or eating problems (Ward-N). Patients were eligible for this study if they were admitted for an acute psychiatric dysfunction (regardless of their clinical diagnosis but excluding organic brain syndromes) and if they were clinically stable after intervention. All participants, upon their agreement, were referred to the principal researcher by four medical teams. Written informed consent was obtained after the procedure was explained.

Eighty-nine participants were approached; 54% were from Ward-P and 46% were from Ward-N. Of all participants, 73% were female, and the averages of age and education were 36 ± 12 years old and 12.8 ± 2.7 years, respectively. The primary clinical diagnoses of these participants included psychotic disorders (*n* = 40, 45%)^1^, bipolar affective disorder (BAD, *n* = 23, 26%)^1^, major depressive disorder (*n* = 15, 17%), eating disorders (*n* = 8, 9%), and others (*n* = 3, 3%). All of the primary clinical diagnoses of the participants were made according to the DSM-IV-TR before their admission. None of the participants were diagnosed with a dissociative disorder. Nine participants did not complete the assessment because of unstable mental status. Data from 80 participants were used in the analysis.

### Instruments

#### Dissociation

Two self-report scales were used. The Dissociative Experiences Scale (DES, Cronbach's alpha = 0.95) comprises 28 items covering absorption, depersonalization-derealization, and amnesia (Carlson and Putnam, 1993). Each item is measured on an 11-point scale from 0 (never) to 100 (always). The DES is viewed as a measure primarily for non-pathological dissociation, as most of the items (20 of the 28) pertain to absorption and cannot differentiate patients with a dissociative disorder from other individuals with non-pathological dissociation (Waller et al., 1996). Some researchers have used an eight-item version (i.e., DES-Taxon) to measure pathological dissociation. However, problematic reliability (Watson, 2003) and validity (Leavitt, 1999; Simeon et al., 2003) were observed possibly due to the small number of items. Therefore, we adopted another instrument developed by the author of the DES (Eve Carlson), the Traumatic Dissociation Scale (TDS, Cronbach's alpha = 0.93) to measure pathological dissociation (Carlson and Waelde, 2000; Carlson et al., 2011). The TDS has 24 items from five subscales (depersonalization, derealization, gap in awareness, gap in awareness and intrusion, and amnesia). Each item is measured on a five-point scale from 0 (not at all) to 4 (more than once a day).

#### Psychosis

Two measures were used to assess psychotic symptoms. The Positive and Negative Syndrome Scale (PANSS) is an interview schedule used to evaluate positive and negative symptoms (Kay et al., 1987). Each item is measured on a seven-point scale from 1 (absent) to 7 (extreme). Schneiderian first-rank symptoms were measured by eleven items (0 = no, 1 = yes) from the Dissociative Disorders Interview Schedule (DDIS) (Ross, 1997).

#### General psychopathology and trauma history

The Symptom Check List-90-Revised (SCL-90-R, Cronbach's alpha = 0.98) is a scale measuring global psychopathology (Derogatis, 1983). The SCL-90-R contains 90 items covering nine scales, including somatization, obsession-compulsion, interpersonal sensitivity, depression, anxiety, hostility, phobic-anxiety, paranoid ideation, and psychoticism. Each item is rated on a five-point scale from 0 (not at all) to 4 (extremely). The total score excluding the paranoid ideation and psychoticism subscales was used. The Brief Betrayal Trauma Survey (BBTS; Cronbach's alphas = 0.76 and 0.79 for the childhood and adulthood subscales, respectively) is a scale measuring trauma history (Goldberg and Freyd, 2006). Twelve items covering diverse traumatic experiences including natural disasters, traffic accidents, witnessing violence, physical assault, and sexual maltreatment in childhood (before 18) or adulthood are rated. Each experience is rated on a three-point scale (never, one to two times, and more than two times). The three-point score was converted into a dichotomous variable with 0 (no) or 1 (yes). Two total scores (childhood trauma and adulthood trauma) were derived.

### Cognitive tasks

#### Intellectual function

A short-form of the Wechsler Adult Intelligence Scale (the 3^rd^ version, WAIS-III) composed of information and block design in conjunction with arithmetic and digit symbol substitution was used to evaluate intellectual function (Blyler et al., 2000; Ringe et al., 2002). The intellectual quotient was used in the analysis.

#### Source monitoring task

On the basis of the rationale adopted in previous self-vs.-other source monitoring tasks (Fisher et al., 2008; Mitchell and Johnson, 2009) and participants' feedback from a pilot test, we developed a version that was composed of common Taiwanese knowledge and was appropriate for the linguistic features of Mandarin Chinese. There were two categories of sentences, one related to occupation and the other to animals. Each sentence was composed of one subject, one verb, and one object (e.g., “Postmen deliver letters.”). The sentences were randomly divided into three sets for the participant-generated condition, the experimenter-provided condition, and the new items of the recognition test.

In the study phase, the sentences of the first and second sets were read aloud by an experimenter, who tapped per character on a table to indicate the length of each sentence. Participants were required to read the sentences aloud again. In the experimenter-provided condition, each sentence had a complete subject. Participants merely repeated the full sentence. In the participant-generated condition, a sentence with an incomplete subject was read aloud. Only the first character of the subject was provided, and the other characters were omitted (most subjects were two-character nouns and the others were three-character nouns). Based on the taps, participants were made aware of the number of omitted characters and attempted to complete each sentence under instruction. The subjects generated by the participants were written down and used in the recognition test. The two types of sentences were presented intermittently. The study phase was completed when the participant successfully generated eight meaningful sentences for the participant-generated condition.

In the test phase, the experimenter read complete sentences aloud from the three conditions. The participants were required to answer whether the sentence had been learned in the study phase. If the sentence was learned previously, they were required to judge whether the subject of the sentence was generated by themselves or was obtained from the experimenter. The first block was the occupation-related sentences, followed by the animal-related sentences. The three types of sentences (experimenter-provided, participant-generated, and new items) were randomly presented. The accuracy rates of recognizing learned and new sentences, which reflected participants' item recognition ability, were computed for each participant. The accuracy rates of judging the sources of the experimenter-provided and participant-generated subjects were computed for each participant to assess their ability in source monitoring.

### Procedure

Assessments were held individually in an interview room of each ward. The principal researcher conducted the assessment including the cognitive evaluation and the diagnostic interview. The principal researcher (CC) was a board-certificated clinical psychologist who had received extensive training on dissociation and psychosis assessment and the use of the instruments and tests. The diagnostic interviews were conducted first, followed by the cognitive tasks and finally the self-report measures. Each participant's interview ratings were cross-validated with medical charts and the clinical staff who took care of the patients. The cognitive tasks began with the intelligence test, the source monitoring task, and then several executive control tasks not reported here^2^. After completing the cognitive tasks, the participants were tested on their memory of the source monitoring task. Participants then completed the self-report instruments.

## Results

Table 1 summarizes the means and standard deviations of the major measures. The relationship found between trauma and dissociation has been reported elsewhere (Chiu et al., 2015), and traumatic experience was considered a covariate in this study.

**Table 1:** **The means and standard deviations of the measures of psychopathology and trauma history**.

**Instruments/scale or subscale**	***M***	***SD***
**DES**		
Total score	23.4	19.4
Absorption	27.8	20.5
DP/DR	21.2	21.4
Amnesia	13.7	20.0
**TDS**		
Total score	39.3	15.7
Depersonalization	6.9	3.0
Derealization	7.7	3.7
Gap in awareness (GAP)	9.5	4.3
Amnesia	5.5	3.1
GAP and intrusion	9.7	3.7
**SCL-90-R**		
Total score	1.2	0.9
**BBTS**		
Childhood trauma	2.1	2.4
Adulthood trauma	2.4	2.6
**DDIS**		
First rank symptom	1.9	2.2
**PANSS**		
Positive symptoms	15.1	6.1
Negative symptoms	11.3	5.7
General psychopathology	34.7	8.0

*DES, Dissociative Experiences Scale; DP/DR, depersonalization/derealization; TDS, Traumatic Dissociation Scale; BBTS, Brief Betrayal Trauma Survey; SCL-90-R, Symptoms Checklist-90-Revised; DDIS, Dissociative Disorders Interview Schedule; and PANSS, Positive and Negative Syndrome Scale. The items corresponding to each subscale are the follows: for the DES (Stockdale et al., 2002), absorption (1, 2, 10, and from 14 to 26), DR/DP (7, 11, 12, 13, 27, and 28), amnesia (3, 4, 5, 6, 8, and 9); for the TDS (Carlson et al., 2011), depersonalization (1, 7, 11, 15, and 20), derealization (2, 6, 10, 16, and 19), gap in awareness (3, 9, 13, 18, and 22), amnesia (5, 14, and 23), gap and intrusion (4, 8, 12, 17, 21, and 24)*.

### Memory performance

We first explored source monitoring performance to examine overall accuracy across participants. The accuracy of recognizing new items was high (*M* = 0.96, *SD* = 0.16), indicating that most participants had low false positives. A 2 (judgment: item, source) × 2 (condition: experimenter-provided, participant-generated) repeated-measures analysis of variance (ANOVA) was conducted. The two-way interaction reached significance [*F*_(1, 79)_ = 51.82, *p* < 0.01, *r*_*contrast*_ = 0.40]. Matched *t*-tests showed a significant difference between the participant-generated and experimenter-provided conditions in item recognition but not in source judgment [*t*_(80)*s*_ = 13.44 and –0.63; *p*s < 0.01 and = 0.53]. Regarding the generation effect on memory (Mulligan, 2001), the accuracy of recognizing participant-generated items was higher than that of experimenter-provided items (*M*s = 0.88 and 0.65, *SD*s = 0.16 and 0.23). Accuracy of source judgment was comparable between the participant-generated and experimenter-provided items (*M*s = 0.82 and 0.84, *SD*s = 0.20 and 0.21).

Pearson's product-moment correlation coefficients (see Table 2) showed that age negatively correlated with recognition of participant-generated items and the source of experimenter-provided items, while intellectual function was positively associated with item recognition and the source of experimenter-provided items. Elder participants showed more errors on both types of source memory, demonstrating the difficulty with associative memory for these participants. Participants of higher intellectual function showed a better memory of information provided by the experimenter, reflecting the fact that they could adopt better encoding strategies. More importantly, source judgment on the participant-generated items did not significantly correlate with demographic variables, intellectual function, or trauma history.

**Table 2:** **Correlation coefficients of self- vs.-other source monitoring performance with demographic variables, global intellectual functions, trauma history, and general psychopathology**.

**Variables**	**Item recognition**		**Source judgment**	
	**Experimenter-provided item**	**Participant-generated item**	**Experimenter-provided item**	**Participant-generated item**
Age	−0.20^Δ^	−0.23^*^	−0.40^**^	−0.08
Gender	−0.05	0.10	0.22^*^	−0.09
Year of education	0.03	0.10	0.18	−0.13
Intellectual function	0.46^**^	0.45^**^	0.44^**^	0.08
Childhood trauma	0.10	−0.02	0.06	−0.05
Adulthood trauma	0.02	<0.01	0.03	−0.10
Global psychopathology	0.16	0.02	0.05	−0.04

** *designated for p < 0.01*,

* *for p < 0.05*,

Δ for p < 0.10;

*intellectual function, intellectual quotients derived from a short-form of the WAIS-III; childhood trauma, the total score of potentially traumatizing events before 18 in the BBTS; adulthood trauma, the total score of potentially traumatizing events after 18 in the BBTS; Global psychopathology, the total score of the SCL-90-R without the psychoticism and paranoia subscale*.

### Dissociation, psychosis, and self-other source monitoring

Table 3 shows the Pearson's product-moment correlation coefficients between dissociation, psychosis, and memory performance. Our results showed that dissociation negatively correlated with source judgment of participant-generated items but not with source judgment of experimenter-provided items or recognition of both types of items. High dissociation was related to the tendency to attribute a target item that was generated by the participants themselves as an item obtained from the experimenter. Importantly, significant correlations were not found with all dimensions of dissociation. Amnesia (Cohen's *d*s = 0.49 and 0.54) and absorption (Cohen's *d* = 0.52) were associated with source attribution bias. In contrast, source misattribution was not associated with depersonalization, derealization, gap in awareness, or gap in awareness and intrusion. Source misattribution was associated with two aspects of dissociation related to memory difficulty and a blurred boundary between the internal and external worlds.

**Table 3:** **Correlation coefficients among dissociation, psychosis, and self- vs.-other source monitoring performance**.

**Variable**	**Item recognition**		**Source judgment**	
	**Experimenter-provided item**	**Participant-generated item**	**Experimenter-provided item**	**Participant-generated item**
DES total score	−0.11	−0.08	−0.06	−0.24^*^
Absorption	−0.10	−0.06	−0.03	−0.25^*^
Depersonalization and derealization	−0.07	−0.02	−0.03	−0.14
Amnesia	−0.14	−0.17	−0.17	−0.26^*^
TDS total score	0.14	−0.01	0.04	−0.15
Depersonalization	0.12	0.05	0.06	−0.13
Derealization	0.14	0.08	0.09	−0.06
Gap in awareness	0.17	−0.01	0.08	−0.10
Amnesia	0.08	−0.06	−0.08	−0.24^*^
Gap in awareness and intrusion	0.09	−0.08	0.004	−0.15
DDIS-Schneiderian	−0.06	−0.13	−0.02	−0.23^*^
PANSS-Positive	−0.14	−0.0004	−0.03	−0.32^**^
PANSS-Negative	−0.10	−0.13	−0.27^*^	0.02

** *designated for p < 0.01*,

* *for p < 0.05*,

*Δ for p < 0.10; DES, Dissociative Experiences Scale; TDS, Traumatic Dissociation Scale; DDIS-Schneiderian, the score of Schneiderian first rank symptoms in the DDIS; PANSS-Positive or PANSS-Negative, the positive or negative symptom subscale of the Positive and Negative Syndrome Scale*.

Regarding psychotic symptoms, the score of positive symptoms and the number of Schneiderian first-rank symptoms negatively correlated with source judgment of participant-generated items (for positive symptoms and Schneiderian first-rank symptoms, Cohen's *d*s = 0.68 and 0.47, respectively) but not with source judgment of experimenter-provided items nor recognition of both types of items. More Schneiderian first-rank symptoms and higher scores on positive symptoms were related to poor source judgment of participant-generated items. In contrast, the total score of negative symptoms correlated with source judgment of experiment-provided items (Cohen's *d* = 0.56), suggesting that higher scores on negative symptoms were associated with the tendency to attribute a target item obtained from the experimenter as an item generated by the participants themselves.

### Covariate analysis

The effects of covariates on the negative correlations between source judgment of participant-generated items and dissociation were explored. Partial correlations were used to test whether the correlations remained significant after the effects of covariates were removed (see Table 4). First, the correlation coefficients were recalculated when the effects of age, gender, year of education, global intellection function, and general psychopathology were removed. All correlations remained significant. Second, the effects of potentially traumatizing events in childhood and adulthood were removed. Again, the correlations remained significant. The critical result concerned the correlations when the effects of psychotic symptoms were removed. The correlations became marginally significant when either the number of Schneiderian first-rank symptoms or the total score of positive symptoms was removed; the correlations remained significant when the effect of negative symptoms was controlled. The same patterns of results were observed when psychotic symptoms were entered as the predicted variable, as the correlation between dissociative and positive psychotic symptoms was mild (*r*_*DES&PANSS*−*positive*_ = 0.23, *p* = 0.04; *r*_*DES&DDIS*−*Schneiderian*_ = 0.27, *p* = 0.02).

**Table 4:** **Partial correlations between the source judgment of participant-generated items and dissociation subscales**.

**Variables of removal**	**DES-absorption**	**DES-amnesia**	**TDS-amnesia**
Demographic variables	−0.25^*^	−0.26^*^	−0.24^*^
Intellectual function	−0.23^*^	−0.24^*^	−0.22^*^
Trauma history	−0.24^*^	−0.24^*^	−0.22^*^
General psychopathology	−0.28^*^	−0.27^*^	−0.23^*^
Schneiderian symptoms	−0.20^Δ^	−0.22^*^	−0.21^Δ^
Positive symptoms	−0.20^Δ^	−0.21^Δ^	−0.21^Δ^
Negative symptoms	−0.25^*^	−0.26^*^	−0.24^*^

* *designated for p < 0.05*,

Δ *for p < 0.10*.

*DES, Dissociative Experiences Scale; TDS, Traumatic Dissociation Scale; demographic variables, age, gender, and years of education; general psychopathology, the total SCL-90-R score without the paranoid and psychoticism subscales; intelligence, intellectual quotients derived from a short-form of the WAIS-III; trauma history, the total scores of potentially traumatizing events in childhood and adulthood; Schneiderian symptoms, the score of Schneiderian first rank symptoms in the DDIS; positive symptoms, the score of the positive symptom subscale of the PANSS; negative symptoms, the score of the negative symptoms subscale of the PANSS*.

## Discussion

The aim of this study was to investigate the relationship among an altered sense of individuality in self-generated representations, psychosis, and dissociation. We hypothesized that deficient self-vs.-other source monitoring may be a sociocognitive dysfunction related to both dissociation and psychosis. The hypothesis was verified in an unspecified clinical sample of acute psychiatric inpatients with various disorders. Our results showed that the tendency to erroneously attribute participant-generated items as experimenter-provided items was associated with dissociative and positive psychotic symptoms, whereas item recognition was not associated with either symptom cluster. Source misattribution of experimenter-provided items correlated with negative symptoms. The associations between dissociative symptoms and source misattribution of self-generated representations could not be fully explained by intellectual function and general psychopathology, suggesting that source attribution error could be a specific cognitive characteristic of dissociation.

Our results have several important contributions. First, the results clearly indicate that source misattribution was linked to dissociative symptoms as well as positive psychotic symptoms. This finding is consistent with our hypothesis that an altered sense of individuality in self-referential representations underlies both psychosis and dissociation. The mild correlations between the measures of dissociation and of psychosis support that the two should be classified as two distinct pathological clusters. However, both were associated with an altered sense of individuality. The pathogenetic process may differ for the two pathological clusters, as the significant correlations with the DES and TDS subscales were reduced but not eliminated after controlling for either Schneiderian first-rank symptoms or positive symptoms. Psychosis and dissociation may be distinct psychopathological clusters with a common sociocognitive vulnerability in the sense of individuality.

Second, differential associations were found for positive and negative symptoms, as revealed in studies comparing source attribution in schizophrenic patients with different symptom profiles (Brébion et al., 2002; Brunelin et al., 2006). The average effect size in five studies that compared schizophrenia patients with normal controls (Vinogradov et al., 1997, 2008; Moritz et al., 2003; Fisher et al., 2008; Wang et al., 2011) was estimated from their results using Cohen's *d*, resulting in a size of 1.17 relating positive symptoms to misattribution of participant-generated items and a size of 0.37 associating negative symptoms to misattribution of experimenter-provided items. Our results showed a smaller effect size in the correlation with positive symptoms (0.68) and a larger effect (0.56) between negative symptoms and source misattribution. The effect sizes of our results are comparable to those obtained from the two studies (Brébion et al., 2002; Brunelin et al., 2006) that compared different subgroups of schizophrenia patients (effect sizes were 0.59 and 0.72, respectively). Our results showed that the relationship between positive and negative symptoms and the two types of source misattribution could be generalized beyond schizophrenia disorders. Positive symptoms such as hallucination and delusion, which reflect over-sensitivity to the external environment, may impair the sense of individuality and lead to misjudging the source of self-referential representations. Negative symptoms such as emotional withdrawal, which reflect insensitivity to the external environment, may also impair the sense of individuality but lead to misjudging experimenter-provided items as self-generated. There may be two sides to the diminished sense of individuality. These findings are congruent with a phenomenon-based formulation that postulates that deficient processing of self-referential information underlies both positive and negative symptoms (Sass and Parnas, 2003; Nelson et al., 2014). The findings are inconsistent with an earlier perspective that stated that source monitoring was a problem of the positive symptoms of schizophrenia (Frith, 1987). Future research may clarify the associations, including the link between various symptom profiles and the two types of source misattribution in schizophrenia as well as the correlation between source misattribution of self-generated items and positive symptoms in other disorders with psychotic features.

Third, the correlation between the source misattribution of self-generated representations was significant only on two subscales of dissociation: absorption and amnesia. It may be surprising that source misattribution was not related to depersonalization and derealization in dissociation. Of note, there is a debate regarding the dissociative nature of depersonalization and derealization, as they may also result from heightened anxiety reactions (Spiegel et al., 2013). However, the results are consistent with the conceptual discussion of the distinction between depersonalization and amnesia and the multi-dimensionality of dissociation (Holmes et al., 2005; Nijenhuis and van der Hart, 2011). Absorption is the tendency to overly immerse oneself into mental activity without awareness of other concurrent external events. We hypothesized that overly immersing oneself may have caused an altered sense of reality, which requires a clear boundary between the self and the outside world. Accordingly, the individual's sense of individuality may have been diminished. Thus, absorption should be related to both types of source misattribution. Interestingly, absorption did not correlate with source attribution of experimenter-provided items. This asymmetry in findings suggests that a blurring of boundaries impairs self-referential representations. As self-referential representations were impaired, source misattribution of participant-generated items was associated with amnesia. The significant correlation between source misattribution and amnesia is of interest. Past laboratory studies conceptualized dissociative amnesia as a consequence of voluntary avoidance, postulating that memory suppression may be a neurocognitive mechanism of dissociators' forgetfulness (Anderson and Green, 2001). However, empirical evidence does not support this contention (McNally et al., 2001; Elzinga et al., 2003; Chiu et al., 2010, 2012a). It is plausible that memorized items are retained rather than suppressed. While recollecting a memory of autobiographical experience, dissociators may misattribute the source as external rather than self-referential. These intriguing questions remain topics for further investigation.

Fourth, the results suggest that trauma cannot explain the relationship between source misattribution and dissociation. After controlling for potentially traumatizing events, the correlations between the source misattribution of self-generated items and dissociation remained significant. This finding is inconsistent with the belief that early trauma may be the major cause of the altered sense of individuality in dissociation (van der Hart and Witztum, 2008; Markowitsch and Staniloiu, 2011). In fact, similar findings have been reported in studies of atypical executive controls in nonclinical dissociative individuals (DePrince et al., 2008; Chiu et al., 2010, 2012a). Together with laboratory studies, a set of aberrant neurocognitive functions including self-relevant sociocognitive functions and executive controls may be characteristics of dissociation. Although these aberrant neurocognitive functions may not directly result from traumatic stress (Chiu et al., 2012b), they may moderate the adaptation to a traumatic experience, leading to pathological trauma memory and the emergence of pathological dissociation (Chiu et al., 2015).

It is noted that one important feature of dissociative patients is the clinical presentation of multiple symptoms including anxiety, mood symptoms, somatization, and psychotic symptoms (Steinberg et al., 2005; Spiegel et al., 2013). Although participants were not diagnosed by the medical teams as having dissociative disorders, comorbidity did exist, and the symptom severity and chronicity of a disorder were not considered in the analysis. Nevertheless, we considered this factor as less likely to contaminate the results for two reasons. First, the association between dissociation and source misattribution of participant-generated items remained significant after controlling for general psychopathology. Second, previous studies have also reported that symptom fluctuation cannot account for source misattribution in psychotic patients (Vinogradov et al., 1997). Future research should adopt a better design to tackle this issue, such as studying source attribution performance between patients with and without dissociative pathology within a mental disorder using formal instruments for diagnosis (i.e., post-traumatic stress disorder, borderline personality disorder, or psychotic disorders)^3^.

Another limitation of this study was that the sample size was small, and most of the participants were receiving medications at the time of testing. As these participants may have taken other medications prior to admission, we did not analyze whether medication at testing time played a role in source misattribution. However, source misattribution in patients with schizophrenia has been shown to be unrelated to medication status (Vinogradov et al., 1997, 2008; Keefe et al., 2002). Future research could recruit non-medicated participants to better control for the effects of drugs. One possibility would be to study nonclinical individuals with high dissociation proneness because absorption has been correlated with source misattribution. Absorption is a normative manifestation of dissociation and characterizes nonclinical dissociators from non-dissociators (Ross et al., 1990a; Carlson, 1994). In addition, traumatic experiences did not account for the association between absorption and the source misattribution of self-generated items. It is plausible that deficient self-other source monitoring is a cognitive endophenotype that underlies both nonclinical and clinical dissociators. Future research may shed light on this intriguing topic.

## Author contributions

CC designed the research project, conducted data collection and analysis, and wrote drafts of the paper. MT, YC, SL, and CL referred eligible patients to participating in the research. YY and HH supervised the research project and joined the writing of the paper. All authors contributed to critical revisions and interpretations of the findings to result in the final manuscript.

## Funding

The preparation of this manuscript is supported by a grant from the National Science Council (NSC 101-2420-H-002-021-DR) and a start-up grant from The Chinese University of Hong Kong to CC, and a grant from the National Science Council to YY (NSC 102-2420-H-002-009-MY2).

### Conflict of interest statement

The authors declare that the research was conducted in the absence of any commercial or financial relationships that could be construed as a potential conflict of interest.

## Footnotes

^1^Twenty of the 40 psychotic cases were diagnosed with schizophrenia, eight with schizoaffective disorder, four with brief psychotic disorder, two with schizophreniform disorder, one with delusional disorder, and finally six with psychotic disorders not otherwise specified. Thirteen of the 23 BAD cases were in a manic episode when administered into the hospital.

^2^Even though the results from the executive controls tasks were not reported here, a preliminary analysis was conducted to examine if executive functions measured by two tasks (i.e., the random number generation test and the set switching test) could account for the findings. The associations between source misattribution of participant-generated items and dissociation remained significant (*rs* = −0.26 to −0.29) when controlling for the performance on the two tasks. Atypical executive functions did not explain the link between source misattribution and dissociation. An additional correlation analysis was conducted to examine whether the subscale scores of the fours IQ subtests, particularly those involving executive functions (the Arithmetic and Block Design), might correlate differentially with the source attribution of participant-generated items. Consistent with the results using the intellectual quotient, no significant correlations were evident between the scores of the four subtests and the source attribution of self-generated items (*rs* = −0.01 to 0.18, *ps* > 0.11).

^3^An additional analysis was performed to explore the observed correlations separately in psychotic and non-psychotic patients. The two groups were formed based on the primary medical diagnosis (schizophrenia spectrum disorders for the psychotic subgroup, other disorders including bipolar disorders, depressive disorders, and anxiety disorders for the non-psychotic group). The correlation coefficients were comparable numerically to the observed correlation in the whole group and were in the same direction (for the absorption and amnesia scales of the DES, *rs* = −0.20 and −0.24, respectively, in the psychotic group, and *rs* = −0.31 and −0.25 in the non-psychotic group). The results should be interpreted with caution because the sample sizes were too small to reliably test significance.
